# Horses’ Teeth

**Published:** 1881-01

**Authors:** 


### REVIEWS.

HORSES' TEETH: A Treatise on their Mode of Development, Physiological Relations, Anatomy, Microscopical Character, Pathology, and Dentistry. Based on the Notes of well-known Odontologists and Veterinary Surgeons. To which is added a vocabulary of the medical and technical words used. By WILLIAM H. CLARKE. 16mo, pp. 262. New York: Published by the Author. Price, $1.50.

This book is a venture in the field of veterinary science which we hope to see more frequently imitated. The book is mainly a compilation, admirably arranged and prepared with great thoroughness of detail. The compiled matter is well edited and condensed, much of it being rewritten. The original matter is deserving of attention, and will, no doubt, be read with interest, as the author ascribes its parentage to C. D. Home, D. V. S. The work contains much besides the matter pertaining to horses' teeth, the teeth of many other animals being described and compared with those of the horse; in fact, the work might be very properly entitled Teeth, instead of "Horses' Teeth." It gives the history of the evolution of the horse from early geological periods, the half-teeth, which the author has named *Remnant Teeth*, they being traced back to the Eocene period, at which time they were functionally developed. This fact throws light on what has been a mystery, and the author appears to have made a discovery. There are eight chapters, devoted to the following topics, viz.: Tooth-germs—The Temporary Dentition—The Permanent Dentition—The Canine Teeth or Tushes—The Remnant Teeth—Dental Cysts and Supernumerary Teeth—Horses' Teeth under the Microscope—The Pathology of the Teeth—The Dentistry of the Teeth—Fractured Jaws—The Teeth as Indicators of Age—The Trigeminus of Fifth Pair of Nerves.

The work as a whole is very commendable, and we feel sure it will find a place in the library of all interested in a thoroughly practical as well as scientific knowledge of horses' teeth, and will be found exceedingly valuable both to the student and practitioner o Comparative Medical Science.

